# Early Accreta and Uterine Rupture in the Second Trimester

**DOI:** 10.7759/cureus.2904

**Published:** 2018-07-01

**Authors:** Joshua A Ronen, Krystal Castaneda, Sara Y Sadre

**Affiliations:** 1 Internal Medicine, Texas Tech University Health Sciences Center of the Permian Basin, Odessa, USA; 2 MS3/Ross University School of Medicine, California Hospital Medical Center, Los Angeles, USA; 3 MS4/Ross University School of Medicine, California Hospital Medical Center, Los Angeles, USA

**Keywords:** maternal fetal medicine, perinatology, microangiopathic hemolytic anemia, fetal heart rate tracing, uterine rupture, fetal demise, fetal ultrasound, placenta accreta spectrum, disseminated intravascular coagulation, uteroplacental insufficiency

## Abstract

The differential diagnosis of third trimester bleeding can range from placenta abruptia to placenta previa to uterine rupture and the placenta accreta spectrum (PAS). However, patients with risk factors such as multiple cesarean sections (c-sections), advanced maternal age (AMA), grand multiparity, and single-layer uterine closure are at greater risk of developing these complications earlier than we would traditionally expect.

This case recounts a 38-year-old gravida 6 preterm 3 term 1 abortus 1 live 4 (G6P3114) at 23 weeks and five days gestational age (GA) with a past medical history of preterm pregnancy, pre-eclampsia, chronic abruptia, three previous c-sections, and low-lying placenta who presented to the emergency department (ED) with vaginal bleeding. Initial workup revealed placenta accreta and possible percreta. The patient was placed on intramuscular (IM) corticosteroids in anticipation of preterm delivery. As soon as the patient was stable, she was discharged home. She presented to a different hospital the next day with the same complaints. Imaging was consistent with accreta and her presentation with abruption. During the hospital stay, the patient went into threatened preterm labor (PTL). At first, we suspected preterm premature rupture of membranes (PPROM) due to apparent pooling of amniotic fluid in the vaginal canal. Upon further work up, the diagnosis was consistent with chronic abruption oligohydramnios sequence (CAOS). Before this could be investigated, her hospital course was complicated by acute abruption and Category III/nonreassuring fetal heart rate (FHR) tracing. The patient underwent an emergency c-section at 26 weeks GA as well as a planned supracervical hysterectomy for desired permanent sterilization. During the operation, the patient suffered a postpartum hemorrhage (PPH) of 4500 mL. She was later discharged home on postoperative day (POD) eight.

## Introduction

Placenta accreta is a well-known cause of third trimester bleeding. Its incidence has markedly increased in the past few decades, highlighting the importance of swift detection and prompt management of this obstetrical emergency upon presentation. The placenta accreta spectrum (PAS) also includes placenta increta and placenta percreta, which are collectively referred to as morbidly adherent placenta. The pathophysiology of morbidly adherent placenta is characterized by abnormal adherence and invasion of the placenta to the superficial and deep layers of the uterine wall [1]. Resnick et al. discuss that although it occurs in 0.2% of all pregnancies, placenta accreta accounts for roughly 79% of abnormal attachment cases while placenta increta and placenta percreta involves deeper invasion account for 14% and 7% of cases, respectively [1]. Although the pathogenesis is not entirely understood, the most common theory involves defective decidualization allowing myometrial invasion of the placenta possibly from previous surgery (e.g., c-sections) or other anatomical factors.  

Patients who are at the highest risk for placenta accreta are those with a placenta previa affecting their current pregnancy and they have a history of multiple c-sections. Placenta previas—placentas overlying the internal cervical os in varying degrees—are also included in the spectrum of differential diagnoses of third trimester bleeding. Prospective studies have examined the incidence of placenta accreta in patients with concurrent underlying previas. Resnick et al. explain that these studies found the frequency of placenta accreta to be increased commensurately with the number of previous c-sections after the first from three to up to 67% (in one c-section to five to six or more c-sections, respectively) in such expectant mothers. First suspicion of placenta accreta is about obstetrical ultrasound (US) when the patient is asymptomatic. No single modality can establish the prenatal diagnosis of placenta accreta with absolute certainty although the diagnosis is more likely between 18 and 20 weeks gestational age (GA) (with imaging evidence of abnormal implantation) in patients with previa or low-lying placenta after one or more c-sections. As per Resnick et al., clinicians should be aware that the placenta will not deliver by normal means as it is deeply embedded within the uterine wall; this will require manual separation which can precipitate severe postpartum hemorrhage (PPH) [2].

Uterine ruptures are often associated with a trial of labor after the cesarean delivery (TOLAC), especially if the prior cesarean delivery involved a classical incision. TOLAC refers to attempted labor during a vaginal birth after (a prior delivery by) c-section (VBAC). As per Landon et al., incidence rates of uterine rupture at term for TOLAC are even higher than in women who undergo elective repeat cesarean delivery (ERCD), 78% versus 22%, respectively [3]. Uterine rupture is a life-threatening pregnancy complication for both the mother and the fetus. All uterine layers are disrupted including the serosa, ultimately leading to decline in maternal and fetal status. The patient will present with vaginal bleeding, sudden or worsening abdominal pain, hemodynamic instability secondary to hemopertioneum, and nonreassuring fetal heart rate (FHR) tracing (Category II or Category III) due to deterioration of fetal status. Clinicians should note that placental abruption is actually the leading diagnosis in pregnant women with acute abdominal pain, bleeding, and Category II or III tracing. It may not be distinguishable from uterine rupture before exploratory laparotomy.

## Case presentation

**Figure 1:**
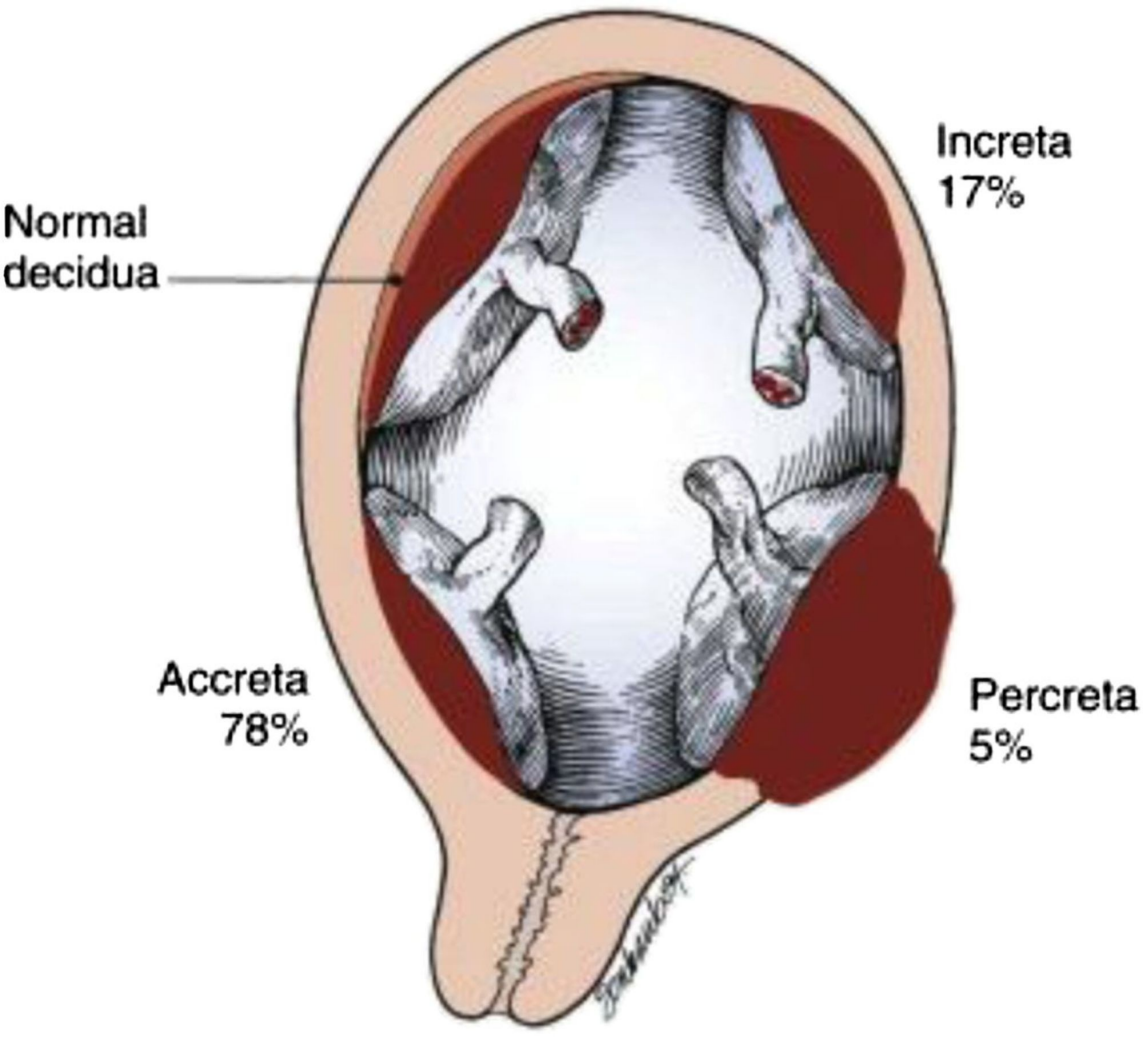
Placenta accreta spectrum. Reference [4] Key: *Accreta: anchoring placental villi attach to the myometrium instead of the decidua *Increta: chorionic villi penetrate into the myometrium *Percreta: chorionic villi penetrate through myometrium to the uterine serosa or adjacent organs (i.e., bladder) This figure was used from Google Images with consent.

This case describes a 38-year-old G6P3114 at 23 weeks and five days GA with chronic abruptia and low-lying placenta who presented to the ED with vaginal bleeding. Her past medical history was significant for preterm pregnancy, preeclampsia, and three previous c-sections. Initial workup revealed placenta accreta and possible percreta (Figure 1) [4]. The patient was placed on IM corticosteroids in anticipation of preterm delivery. As soon as the patient was stable, she was discharged home. She presented to a different hospital the next day with the same complaints.

**Figure 2:**
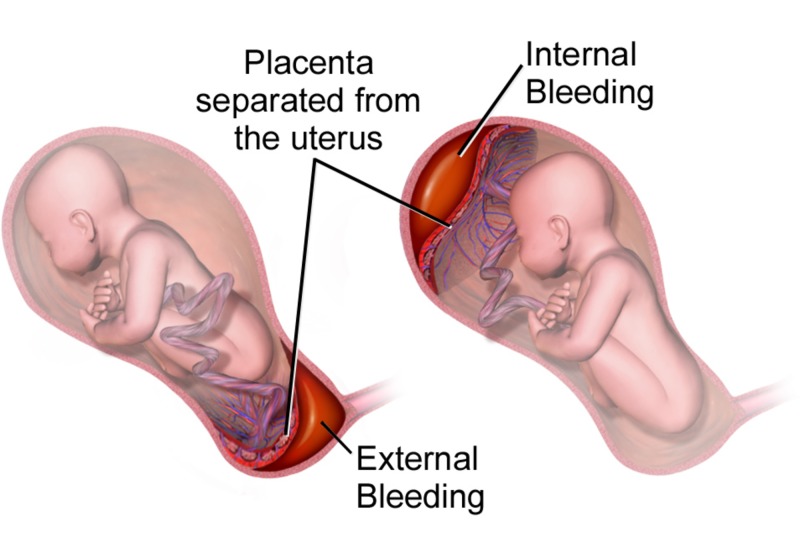
Placental abruption. Reference [5] This figure was used from WikiJournal of Medicine with consent.

The maternal fetal medicine (MFM), neonatal intensive care unit (NICU), and anesthesia teams were consulted on her case due to the concern of placenta accreta. A magnetic resonance imaging (MRI) was done and was significant for loss of the decidual line along the right lateral anterior uterus with myometrial thinning along the region of her previous c-section scar. There was no evidence of percreta on the MRI. Of note, her bedside transvaginal ultrasound (TVU) showed placenta accreta with low-lying anterior placenta with a short cervix and funneling, but ruled out placenta previa. Still, the patient continued to have vaginal bleeding presumably from chronic abruption (Figure 2) [5]. She was transferred back and forth between labor and delivery (L&D) unit and the maternal fetal care unit (MFCU) with threatened PTL.

A few days later, the patient was complaining of leakage of fluid and while on sterile speculum examination (SSE), there was vaginal pooling. Standard diagnostic strategies (nitrazine blue testing and presence of ferning on microscopy of fluid) were used to determine whether the fluid was indeed amniotic and came up positive. Treatment for PPROM was started which included antibiotics as well as rescue steroids. Upon further assessment, it was found that amniotic fluid index (AFI) >7 cm. Subsequent amniotic fluid exam via repeat US the next day was consistent with oligohydramnios. It was thought that her low-lying placenta could have also caused retroplacental blood to accumulate. But, based on the finding of oligohydramnios, chronic abruptia oligohydramnios sequence (CAOS) was more likely the diagnosis than PPROM. Before any further evaluation could be done, the patient went into PTL that night, which ultimately was spontaneously arrested. Her PTL was then complicated by presumed uterine rupture at the site of her previous c-section, as indicated by deterioration of her FHR tracing to Category III.

The patient received general anesthesia for an emergent c-section in the setting of uterine rupture at 26 weeks GA. She then underwent a planned supracervical hysterectomy. The surgery was complicated by PPH as the patient's estimated blood loss (EBL) was about 4500 mL. She received one unit of packed red blood cells (pRBCs) preoperatively the night before, seven units intraoperatively, and two units postoperatively. She was also given four units of fresh frozen plasma (FFP), one unit of platelets, and one unit of cyroprecipitate. She was stable postoperatively and was discharged on POD eight in stable condition.

## Discussion

The patient presented to the hospital at 23 weeks and five days with vaginal bleeding where she was diagnosed with placenta accreta and possible percreta. An accreta suggests that anchoring chorionic villi of the placenta attach to the myometrium instead of the decidua (Figure 1) [4]. The cause is unknown although the most common theory points toward defective decidualization from previous surgery or anatomical factors (i.e., endocervix, lower uterine segment, etc.) that allow the placenta to attach directly to the myometrium. Resnick et al. found that this theory has been already supported with evidence that 80% of accreta patients have had prior history of c-section (consistent with this case), dilation and curettage, or myomectomy [1]. Furthermore, prospective studies have examined the incidence of placenta accreta in patients with underlying placenta previa. They found that the frequency of placenta accreta increased commensurately with the number of c-sections after the first from 3% to up to 67% (one c-section to five to six or more, respectively) in such expectant mothers. The patient in this case had three prior c-sections and a low-lying placenta (one of the several variants of placenta previa) identified, bringing the expected frequency of accreta in patients like her to a noteworthy 40% [1]. Therefore, her risk to develop an accreta at this point of time in her reproductive years was increased. Multivariable analyses challenged the theory Resnick et al. referenced, stating that placenta previa is more of an independent risk factor for accreta than prior uterine surgery (odds ratio 54 versus 1.5 at a 95% confidence interval) [1].

**Figure 3:**
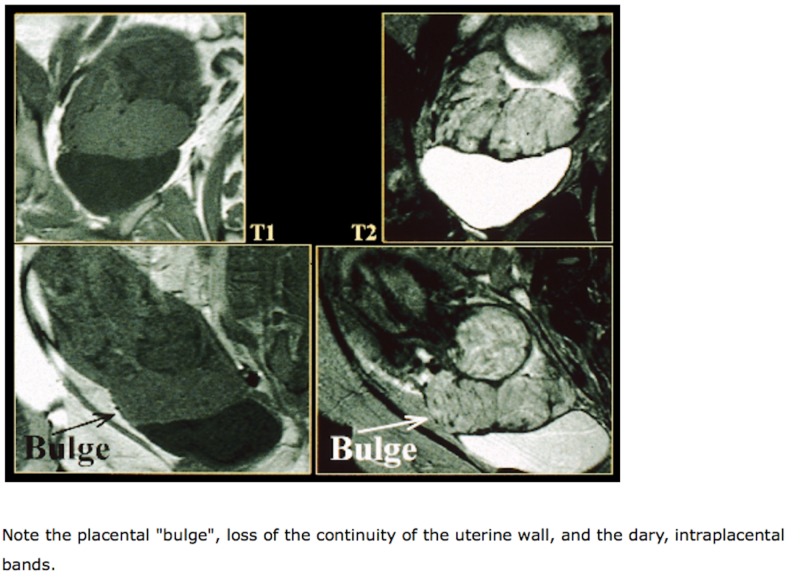
Magnetic resonance imaging suggestive of placenta accreta. Reference [6] This figure was used from UpToDate with consent.

**Figure 4:**
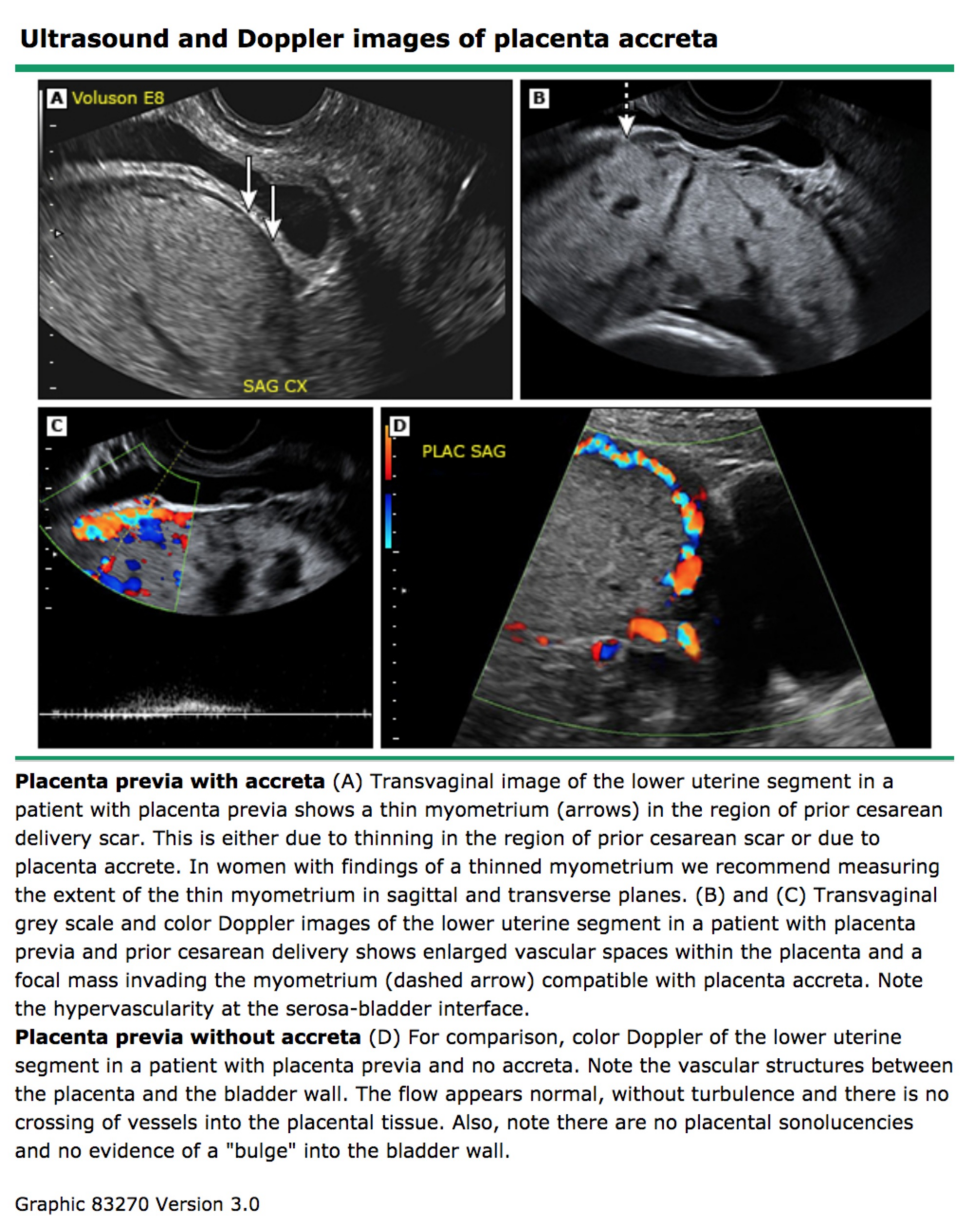
Ultrasound and Doppler images of placenta accreta. Reference [7] This figure was used from UpToDate with consent.

The diagnosis of placenta accreta is typically made in the second trimester around 18-20 weeks, prior to the onset of symptoms in the third trimester. To make the diagnosis, imaging modalities and blood tests can be used. Elevated maternal serum alpha fetoprotein (msAFP) in the second trimester (>2.0-2.5 multiples of the median) supports an US-based diagnosis of placenta accreta but is not useful on its own. The imaging modalities typically used include two-dimensional transabdominal and transvaginal US, color Doppler US, three-dimensional power Doppler, and magnetic resonance imaging (MRI) (Figures 3-4) [2, 6-7]. While usage of any of these imaging modalities has its own benefits, Resnick et al. highlight transabdominal and transvaginal US as the most sensitive (90.7%) and specific (96.7%) studies to evaluate placental position and implantation [2]. The accuracy of first trimester sonographic diagnosis of placenta accreta is still unclear, but second trimester findings include anechoic areas and an irregular placental-myometrial interface. Additional second and even third trimester sonographic findings, touted to be the most common, include loss of placental homogenicity which is replaced by intraplacental sonolucent spaces such as venous lakes or placental lacunae adjacent to the involved myometrium as well as hypervascularity of the serosa-bladder wall interface (disruption of the "bladder line") (Figure 4) [7].

**Figure 5:**
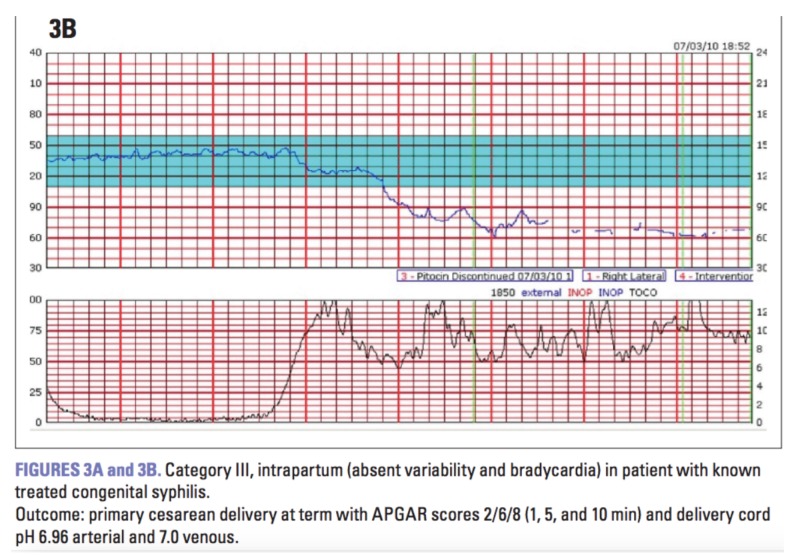
Example of Category III fetal heart rate tracing. Reference [8] This figure was used from The Female Patient with consent.

Transitioning to uterine rupture, incidence is higher in women with previous c-sections (who undergo induction) than in those with spontaneous delivery. Due to the fact that the rupture is increased by the use of prostaglandins, Landon et al. states that the American College of Obstetrics and Gynecologists (ACOG) has advised against the use of misoprostol in women with previous history of c-sections [4]. The risk of rupture is 2.45%. Oxytocin presents a 1.1% risk of rupture yet is not contraindicated when compared to misopristol. He goes on to explain how possible risk factors for uterine rupture include advanced maternal age (AMA), GA > 40 weeks, birth weight >4000 g, single-layer uterine closure, and more than one previous c-section [4]. Measurement of the thickness of the residual myometrium in the lower segment of the uterus at the site of previous c-section can help to determine the risk of uterine rupture. Clinical signs of rupture, as per Landon, et al., include hemodynamic instability, vaginal bleeding, abdominal pain, weakening contractions, loss of station of the fetal presenting part, and FHR abnormalities [4]. Intrapartum FHR tracings help assess the status of fetal cardiovascular well-being. The FHR abnormalities refer to sudden development of consistently Category II or III tracings. A Category I tracing indicates reassuring fetal status while a Category III tracing (Figure 5) indicates nonreassuring fetal status and increased risk of fetal acidemia, necessitating expeditious c-section [8]. Patients with Category II tracings should be managed expectantly and preparations should be made for urgent delivery. As a diagnosis of placental abruption cannot be distinguished from uterine rupture before exploratory laparotomy, clinicians should have a high index of suspicion in patients who present with the aforementioned signs and symptoms. A Category II FHR tracing in the hour preceding rupture increases the likelihood of this intraoperative diagnosis, as Landon et al. emphasize that further deterioration to Category III will lead to prompt c-section [4]. 

Given the patient’s history of chronic placental abruption, equivocal testing for PPROM with Amnisure and SSE, and reduction in AFI, she was given the diagnosis of CAOS. According to Elliott et al., the condition is defined by the following criteria: (1) clinically significant vaginal bleeding in the absence of placenta previa or other identifiable source of bleeding; (2) amniotic fluid volume initially documented as normal; (3) oligohydramnios with an AFI less than or equal to five eventually developing without concurrent evidence of ruptured membranes [9]. The CAOS occurs in pregnancies complicated by placenta abruptia and if it develops, the mean GA at delivery is 28 weeks (i.e., preterm). Attending physicians should be aware that although this condition is rare, according to Kurata et al. it does present risk of lung injury to the infant, which is a major clinical concern [10]. The MRI studies describe CAOS pathophysiology as chronic separation of the placenta, exposing its peripheral veins thus resulting in a marginal hematoma distributed extensively along the decidua. There were no blood-derived products in the amniotic fluid on the patient’s imaging results, which would otherwise show high signal intensity and help confirm the diagnosis on T1-weighted imaging. As Kurata et al. goes on to explain, however, as a consequence of the chronic venous bleeding from the placenta, blood-derived products can leak into the amniotic fluid and the fetus can aspirate them consequently yielding lung injury [10].

The MFM specialists still agree that there are several risk factors that increase the risk for placental abruption alone: substance abuse (cocaine/tobacco), AMA, uncontrolled hypertension (HTN)/preeclampsia, prior history of abruption (7x), asthma (1.2x), and blunt abdominal trauma (6x). Previous abruption confers the strongest risk for future occurrence with recurrence risks at 10 up to 93 times higher according to prior studies. Smoking increases risk by 2.5 times (40% for each pack per day smoked) and has a synergistic effect with HTN while HTN alone increases risk by five times [11].  Epidemiologically, Kurata et al. explain that abruptia occurs in 1% of pregnancies with two-thirds of cases being severe. Some 40%-60% occur <37 weeks GA and 14% <32 weeks GA [10]. The diagnosis of placenta abruptia is reserved for pregnancies over 20 weeks GA. It is a common cause of mild to moderate third trimester bleeding and characterized by complete or partial premature placental detachment prior to the delivery of the fetus. The FHR tracings will become nonreassuring (Category II or III) and the patient will also experience bleeding and abdominal/back pain as well as uterine tenderness. As per Ananth et al. the perinatal mortality rate appears to be declining, but it is still 20 times higher in comparison to pregnancies without abruption (12% versus 0.6%) [11]. Nonetheless, 10%-20% of abruptia cases end up being preterm births either from PTL or PPROM with scant or no vaginal bleeding. This is termed a "concealed abruption," difficult to quantify and thus ominous retroplacental hematoma or clot that cannot escape through the cervix and vagina. Even a small amount of bleeding in pregnant women with these symptoms should warrant prompt evaluation.

**Figure 6:**
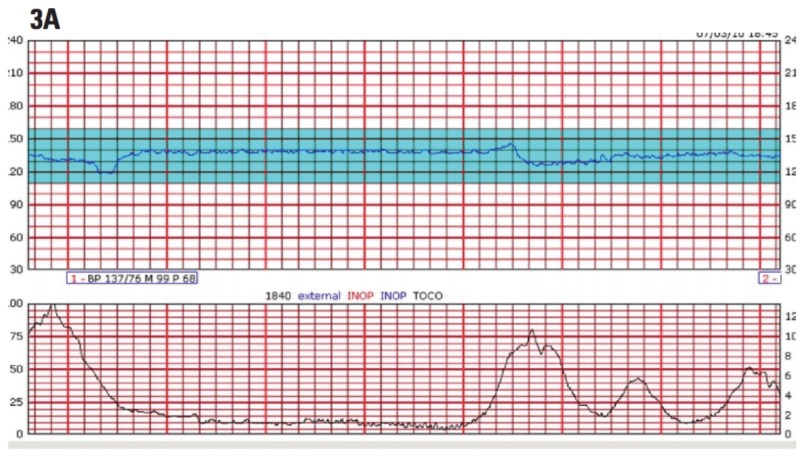
Example of fetal heat rate exhibiting late decelerations. Reference [8] *Late decelerations indicate uteroplacental insufficiency, poor circulation of oxygen, nutrients, and metabolic byproducts between the mother and fetus *in utero*. Fetuses exhibiting prolonged late decelerations warrant expeditious delivery via caesarean section to circumvent maternal and fetal morbidity. This figure was used from The Female Patient with consent.

Pathogenesis of placenta abruptia as pictured in Figure 2 involves rupture of maternal vessels in the decidua basalis where they interface with anchoring villi of the placenta—blood accumulates and splits the decidual-placental interface leading to complete or near complete placental separation. This is evidenced by findings of retroplacental hematoma on transabominal US (sensitivity 25%-60%, PPV 88%) [11]. Ananth et al. note that the presence of the following findings in symptomatic patients can improve pretest probabilities in favor of the diagnosis: subchorionic collections of fluid, echogenic debris in the amniotic fluid, and thickened placenta that shimmers with maternal movement ("jello" sign) [11]. Note that the absence of the hematoma does not exclude acute severe abruption because blood may not collect behind the uterus. The consequence of such pathophysiology, however, is uteroplacental insufficiency (and subsequent growth restriction if chronic) to the fetus, excessive blood loss, microangiopathic hemolytic anemia (MAHA), and disseminated intravascular coagulation (DIC) in the mother (if the abruptia is severe). More than 50% of fetal demise cases are stillborn due to intrauterine asphyxia in such settings [11]. These abnormalities, including elevation of msAFP or hCG and decreased PAPPA-A or unconjugated estriol (E1) (10-fold increased risk of subsequent abruption), strongly support the clinical diagnosis as per Ananth et al. [11]. Patient mortalityl depends on the severity of the placental separation, and whether it becomes severe and exceeds 50%. Oyelese et al. caution that laboratory findings suggestive of mild DIC need to be interpreted with caution as normal pregnancy is a hypercoagulable state—there is an increase in concentration of almost all of the coagulation factors and a mild decline in the platelet count [12]. Sections of the placenta that remain attached cannot compensate for the surface area of gas exchange that has been lost in the sections that have separated, thus the FHR tracing will inevitably show late decelerations (Figure 6) [8]. If missed, prolonged late decelerations can result in fetal bradycardia and fetal demise. Nonetheless, Ananth et al. have identified that DIC occurs in 10%-20% of severe abruptions with the death of the fetus [11]. All in all, MFM specialists believe that placenta abruptia is one of the most common causes of fetal demise itself.

Management

The primary aim of placenta accreta is to reduce the risk of massive hemorrhage, which is the most common complication. The first step in management is to have a multidisciplinary care team on board, retrieve informed consent early on, and have a scheduled delivery around 34-35 weeks GA at a well-equipped facility. The probability of complications over the hospital course can be decreased by having a planned c-section compared to an emergent one. Given the risks of massive hemorrhage during attempted placental removal if accreta is present, Resnick et al. believe that recommending a cesarean hysterectomy based on imaging findings is the most reasonable and safest approach to management [3]. Still, however, the use of balloon angioplasty in lieu of hysterectomy should be considered. Baseline hemoglobin levels should be obtained in advance of surgery, if possible, in anticipation of the need to manage massive hemorrhage intraoperatively. Delivery should ideally be done on a unit with 24-hour access to a full blood bank. If the patient desires future childbearing/fertility after extensive counseling about the risks, then discussion about uterine conservation should be done. Uterine conservation with placental resection will depend upon the severity of the accreta, whether it is focal, fundal, or posterior. The patient will require IV access, thromboembolism prophylaxis (PPX), blood products, antenatal corticosteroids, and bladder catheterization. Anesthesia can range from epidural, with or without spinal, to general anesthesia or a combination of both. Balloon catheters may be placed into the internal iliac arteries and inflated intermittently during the hysterectomy. They can also be used for embolization of persistent bleeding.  Postoperative care should include close monitoring of coagulopathy, anemia, thromboembolism, and organ dysfunction. Patients who have experienced a focal accreta or incidentally found accreta are at risk for retained placental tissue. These patients should be managed as such. In cases where the patients are able to preserve their uterus after having placenta accreta, they should undergo counseling for future pregnancies as recurrence rates range from 25% to 35%.

**Figure 7:**
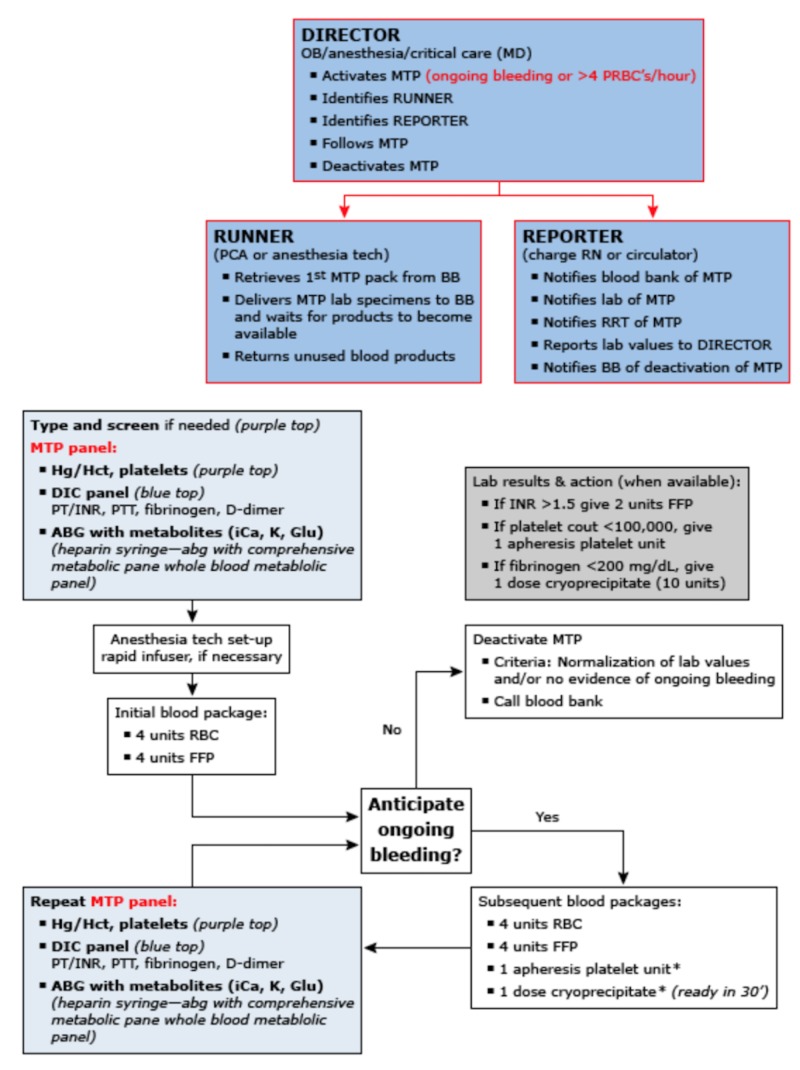
Massive transfusion protocol. Reference [13] This figure was used from UpToDate with consent.

The management of placental abruption is dependent on several factors, including: maternofetal hemodynamic status, EBL, and GA at presentation. In the L&D unit, maternal and fetal vital signs should be continuously monitored. Normotension may mask hypovolemia if the mother has chronic hypertension or pregnancy-induced hypertension. Consequently, the risk of uteroplacental insufficiency looms should maternal blood volume decline unchecked. Upon admission to the emergency room, Oyelese et al. recommend the following orders: complete blood count (CBC), red blood cell type and cross-match (in anticipation of the need for transfusion), coagulation profile, complete metabolic profile (as the patient is at high risk of acute renal insufficiency secondary to blood loss), and urine toxicity [12]. As with any patient who is profusely hemorrhaging, two peripheral large-bore IVs should be started immediately. Lactated Ringer’s (LR) is preferred for volume resuscitation in this patient as well as for maintaining a urine output > 30 mL/hr. If EBL exceeds 500-1000 mL, transfusion of blood products should be initiated. Massive transfusion protocol (MTP) should be initiated if the patient has lost greater than four units (2 L) of blood (Figure 7) [13]. Dependent on the fetus’s GA, Oyelese et al. point out that arrangements for infusion of antenatal corticosteroids, tocolytics, Group B Streptococcus (GBS) PPX (should the patient have tested positive at her 35 week screening), and magnesium sulfate (MgSO4) should be made (if <32-34 weeks GA) [12]. The typical hospital stay lasts a minimum of 48 hours until FHR tracings and US exams are reassuring and the patient is asymptomatic.

 

In anticipation of an increasing amount of blood loss, clinicians should be wary of imminent deterioration of a reassuring FHR tracing (Category I) as well as extravasation of blood into the myometrium (Couvelaire uterus). Studies show in this particular setting that decision-to-delivery time of less than 20 minutes is associated with better outcomes than a 30-minute interval. Category II tracings should be managed expectantly. As such tracing is at risk of worsening to a Category III (Figure 5) in the setting of acute abruption, close monitoring should be prioritized and preparations should be made for urgent delivery of the fetus [8]. The determination for delivery is made based on GA, cervical dilation, and the current maternofetal condition. Should the mother become hemodynamically unstable or develop signs and symptoms of significant coagulopathy, immediate c-section has the greatest mortality benefit. If the abruption was severe enough and a Couvelaire uterus develops, Oyelese et al. warn that the uterus can become atonic and the risk of PPH rises significantly [12]. Intravenous oxytocin and manual uterine massage can be used acutely in this scenario. Nevertheless, the patient is at risk of DIC [fibrinogen < 200 mg/dL, elevated fibrinogen degradation products (FDP), and elevated d-dimer] and exsanguination which can result in multisystem organ failure (MSOF) if the uterine atony is not aggressively managed. Delivery should be scheduled for 37-38 weeks because of the increased risk of stillbirth. Vaginal delivery is preferred if there are contraindications to c-section. The only reason to deliver the fetus prior to even early term (37 weeks) is if additional complications such as fetal growth restriction, preeclampsia, premature rupture of membranes, Category III tracing, or recurrent maternal abruption exist. Oyelese et al. demonstrate, however, that most patients are delivered at 34-36 weeks GA as the risk of worsening placental separation and neonatal morbidity remains [12]. Partial abruption can progress to total abruption suddenly and without warning.

Management of uterine rupture in patients who are not in labor includes undergoing imaging studies [US, MRI, and/or computed tomography (CT)]. They may be done as part of the routine follow-up in patients at risk or during a trauma evaluation. These studies may show disruption of the myometrium, extrauterine fluid-distended fetal membranes, free peritoneal fluid, anhydraminos, an empty uterus, fetal parts outside of the uterus, fetal demise, or pneumoperitoneium (signs of visceral perforation). While these imaging studies may guide management, Landon et al. emphasize that FHR abnormalities, maternal hemodynamic instability, and severe abdominal pain usually require urgent delivery [4]. Hemodynamically unstable patients are stabilized with fluids and blood transfusions and are then prepared for c-section. Abdominal incision options include Pfannenstiel (lower uterine/bikini line) and midline (classical) incision. Pfannanstiel incision has limitations as it only provides good exposure to the lower uterine segment and pelvis while the midline incision provides better exposure for a more thorough abdominal exploration. Midline incision exposes the uterine fundus which will extend above the umbilicus by late second trimester. In the event uterine rupture is discovered during laparotomy, Landon et al. explain that the uterus may be saved through rapid primary single or double-layer closure with a delayed absorbable suture [4]. If the uterine defect cannot be repaired in the setting of uncontrollable hemorrhage, hysterectomy is indicated. With subsequent pregnancies, a scheduled c-section is highly recommended before the onset of labor. Although early rupture of the uterus is rare, Faguer and Endres et al. emphasize that it should not be discounted [14-15]. Faguer recounts the case of a patient at the 22nd week of her third pregnancy who presented with internal hemorrhage that required immediate exploratory laparotomy and subsequent hysterectomy to achieve hemostasis. Faguer concludes that ruptures are more likely to occur in a uterus with an anterior scar from a prior c-section [14]. In Faguer's defense, Endres et al. believe that uterine rupture should be considered in the differential diagnosis of severe abdominal pain in the early second trimester, especially if the patient underwent prior c-sections involving classical incision [15].

## Conclusions

Early detection, diagnosis, and proper management are critical in the setting of placenta accreta and/or uterine rupture to decrease the likelihood of clinical deterioration and maternofetal morbidity and mortality. Adverse outcomes in similar cases have included neonatal periventricular leukomalacia, stillbirth secondary to intrauterine asphyxia, and preterm delivery (<37 weeks GA). To help reduce morbidity and mortality in the setting of placenta accreta, abruption, and/or uterine rupture, MFM and obstetrical specialists should continue to carry a high index of suspicion in patients presenting with similar findings suggestive of any of the aforementioned diagnoses especially during the second trimester.
